# On the Optimization and Self-Tensoring Construction of Nonlinear Entanglement Witnesses

**DOI:** 10.3390/e28060608

**Published:** 2026-05-28

**Authors:** Juan Yu, Lei Li, Ming Li, Shu-Qian Shen

**Affiliations:** College of Science, China University of Petroleum, Qingdao 266580, China

**Keywords:** entanglement detection, entanglement witness, local measurement

## Abstract

Nonlinear entanglement witnesses constructed from multiple linear entanglement witnesses and multiple copies of quantum states have recently been proposed as a powerful tool for entanglement detection. In this work, we show, via an explicit counterexample, that the fineness of linear witnesses generally fails to transfer to their tensor-product nonlinear counterparts. For the canonical family of nonlinear witnesses in the form of (αI−L)⊗(βI−T), we rigorously prove that the optimal nonlinear witness is uniquely attained with weakly optimal parameters of $\alpha =\lambda_{\max}\left(L\right)$ and $\beta =\lambda_{\max}\left(T\right)$. Meanwhile, we analytically demonstrate that the self-tensor products of two representative linear witnesses fail to detect any entangled state. The question of whether a nonlinear entanglement witness capable of detecting entanglement can be constructed by tensoring a linear witness with itself remains open.

## 1. Introduction

Quantum entanglement is a cornerstone of quantum physics. As a physical resource, it is widely applied in quantum computing, quantum communication, and quantum cryptography [1,2]. Effective and reliable entanglement detection therefore becomes particularly crucial. However, entanglement detection is an NP-hard problem [3]. A variety of methods for entanglement detection have been proposed (see, e.g., [4,5] for comprehensive surveys). Most of them require quantum state tomography [6], which is extremely difficult in high-dimensional quantum systems. Entanglement witnesses (EWs) [7] offer a practical and efficient method for experimentally detecting entanglement, avoiding the need for full quantum state tomography.

An EW [7] is a Hermitian operator that takes non-negative expectation values for all separable (unentangled) states while producing negative values for at least one entangled state. Geometrically, an EW corresponds to a hyperplane in the space of quantum states, which separates a subset of entangled states from the convex set of separable states. Thus, EWs are also referred to as linear EWs. Various approaches for constructing linear EWs have been developed (see [8] for a comprehensive survey).

Nonlinear modifications of EWs have been widely explored. In [9], a general method to enhance a given linear EW by adding a quadratic nonlinear term was proposed; the proposed approach can be iterated to generate a sequence of increasingly powerful nonlinear witnesses. In [10], the authors introduced accessible nonlinear EWs that can be evaluated using precisely the same measurement data as the original linear witness. Linear witnesses [11] constructed from local orthogonal observables were further nonlinearly improved and optimized in [12,13]. In [14], a nonlinear improvement for arbitrary 2⊗d EWs was presented, incorporating the entanglement detection inequalities reported in [15,16] as special cases.

Recently, Rico and Huber [17] put forward a class of nonlinear entanglement detection schemes by employing multiple EWs or positive operators, along with copied states. Some illustrative examples show that certain entangled states that cannot be detected by linear EWs can be successfully identified by the corresponding nonlinear method. Analogous strategies were also developed in [18]. In [19], Rico constructed both linear and nonlinear EWs by tensoring and taking partial traces of existing states and witnesses. However, a theoretical analysis for nonlinear EWs in [17] comparable to that for linear EWs is still absent. The aim of this paper is to derive several theoretical results for nonlinear EWs [17], laying a theoretical foundation for their construction and application.

The remainder of this paper is organized as follows. After reviewing the necessary preliminaries in Section 2, we investigate the optimization of nonlinear EWs in Section 3. In Section 4, we present a conjecture that nonlinear EWs capable of detecting entanglement cannot be constructed from identical EWs. Finally, we conclude in Section 5 with some remarks.

## 2. Preliminaries

A state $\rho_{AB}$ on $C^{d}⊗C^{d}$ is separable [20] if and only if it can be expressed as

$$\rho_{AB}=\sum_{i}p_{i}\rho_{i}⊗\sigma_{i},$$

where $p_{i}>0$ and $\rho_{i}$ and $\sigma_{i}$ denote the pure states of the *A* and *B* subsystems, respectively. An observable *W* acting on $C^{d}⊗C^{d}$ is an entanglement witness (EW) [21] if it satisfies the following:

**(i)**  *W* is block-positive, i.e., tr(Wρ)≥0 for any separable state ρ on $C^{d}⊗C^{d}$;
**(ii)**  There exists at least one entangled state σ on $C^{d}⊗C^{d}$ such that tr(Wσ)<0.

Assume that *W* and *V* are EWs acting on $C^{d}⊗C^{d}$. If any entangled state detected by *V* can also be detected by *W*, then *W* is said to be finer than *V* [22]. EW *W* is called optimal if there is no other EW that is finer than *W*. It was shown in [22] that EW *W* is optimal if and only if W−P is no longer block-positive for any non-zero positive operator *P*. According to [23], any EW acting on $C^{d}⊗C^{d}$ can be written as

(1) W=λI−L,

where *L* is a positive operator and λ is a real number satisfying $\lambda \leq \lambda_{\max}\left(L\right)$ with

$$\lambda_{\max}\left(L\right)=\underset{|\alpha \beta \rangle }{\sup}\langle \alpha \beta |L|\alpha \beta \rangle .$$

If λ is chosen to be $\lambda_{\max}\left(L\right)$, then the EW *W* is called weakly optimal [24]. It should be noted that the optimal EW is necessarily weakly optimal, but the converse does not hold.

In [17], Rico and Huber proposed a systematic method for nonlinear entanglement detection using multi-partite witnesses and multiple copies of states. Specifically, there exist EWs or positive operators *W* and *V*, together with a state ρ such that

$$\mathrm{tr}\left(W_{A_{1}B_{1}}⊗V_{A_{2}B_{2}}\rho_{A_{1}A_{2}}⊗\rho_{B_{1}B_{2}}\right)<0,$$

while

tr(Wρ)≥0,tr(Vρ)≥0.

In other words, the entanglement of ρ can be detected by the nonlinear EW expressed as $W_{A_{1}B_{1}}⊗V_{A_{2}B_{2}}$ but not by *W* or *V* individually. Similar to linear EWs, we provide the following definition.

**Definition 1.** 
*If every entangled state detectable by the nonlinear EW expressed as $W_{A_{1}B_{1}}⊗V_{A_{2}B_{2}}$ can also be detected by the nonlinear EW expressed as $M_{A_{1}B_{1}}⊗N_{A_{2}B_{2}}$, then $M_{A_{1}B_{1}}⊗N_{A_{2}B_{2}}$ is said to be finer than $W_{A_{1}B_{1}}⊗V_{A_{2}B_{2}}$.*

## 3. Optimization of Nonlinear EWs

By analogy with the optimization of linear EWs, we are naturally led to the following problem.

**Problem 1.** 
*Suppose that the EWs $W_{A_{1}B_{1}}$ and $V_{A_{2}B_{2}}$ are finer than $M_{A_{1}B_{1}}$ and $N_{A_{2}B_{2}}$, respectively. Is the nonlinear EW expressed as $W_{A_{1}B_{1}}⊗V_{A_{2}B_{2}}$ finer than the nonlinear EW expressed as $M_{A_{1}B_{1}}⊗N_{A_{2}B_{2}}$?*

We now provide an example to illustrate that this is not generally true. The following two-qubit EWs were employed in [17]:

$$\begin{array}{cc} W & =I-X⊗X-Z⊗Z,V=|\phi^{+}\rangle {\langle \phi^{+}|}^{\Gamma }, \end{array}$$

where $|\phi^{+}\rangle$ denotes the Bell state, i.e., $|\phi^{+}\rangle =\frac{1}{\sqrt{2}}\left(|00\rangle +|11\rangle \right)$. Consider another Bell state mixed with white noise:

$$\rho_{p}=p|\phi^{-}\rangle \langle \phi^{-}|+\frac{1-p}{4}I,$$

where 0≤p≤1 and $|\phi^{-}\rangle =\frac{1}{\sqrt{2}}\left(|00\rangle -|11\rangle \right)$. Define M=W+P, where *P* is the positive operator given by [18]:

$$P=\frac{1}{8}\left(\begin{array}{cccc} 1 & 0 & 0 & -1\\ 0 & 4 & -4 & 0\\ 0 & -4 & 4 & 0\\ -1 & 0 & 0 & 1 \end{array}\right).$$

The nonlinear EW expressed as W⊗V detects entanglement of $\rho_{p}$ for 0.7072≤p≤1, while M⊗V detects entanglement for 0.6989≤p≤1. Thus, even though linear EW *W* is finer than *M*, the nonlinear EW expressed as W⊗V is not finer than M⊗V.

However, for nonlinear EWs constructed from linear witnesses in the form of (1), we can derive explicit optimization results. The following lemma is needed.

**Lemma 1.** 
*Let $W_{A_{1}B_{1}}$ be an EW and $V_{A_{2}B_{2}}$ be an EW or a positive operator acting on $C^{d}⊗C^{d}$. If $M_{A_{1}B_{1}}=W_{A_{1}B_{1}}-\tau I$ is also an EW for some τ>0, then $M_{A_{1}B_{1}}⊗V_{A_{2}B_{2}}$ is finer than $W_{A_{1}B_{1}}⊗V_{A_{2}B_{2}}$.*

**Proof.**  For an arbitrary state $\rho =\rho_{A_{1}A_{2}}=\rho_{B_{1}B_{2}}$ on $C^{d}⊗C^{d}$, we obtain

$$\begin{array}{cc} \; & \mathrm{tr}\;\left(M_{A_{1}B_{1}}⊗V_{A_{2}B_{2}}\rho_{A_{1}A_{2}}⊗\rho_{B_{1}B_{2}}\right)\\ \; & =\mathrm{tr}\;\left(W_{A_{1}B_{1}}⊗V_{A_{2}B_{2}}\rho_{A_{1}A_{2}}⊗\rho_{B_{1}B_{2}}\right)-\tau \mathrm{tr}\;\left(I⊗V_{A_{2}B_{2}}\rho_{A_{1}A_{2}}⊗\rho_{B_{1}B_{2}}\right)\\ \; & =\mathrm{tr}\;\left(W_{A_{1}B_{1}}⊗V_{A_{2}B_{2}}\rho_{A_{1}A_{2}}⊗\rho_{B_{1}B_{2}}\right)-\tau \mathrm{tr}\left(V_{A_{2}B_{2}}\rho_{A_{2}}⊗\rho_{B_{2}}\right)\\ \; & \leq \mathrm{tr}\;\left(W_{A_{1}B_{1}}⊗V_{A_{2}B_{2}}\rho_{A_{1}A_{2}}⊗\rho_{B_{1}B_{2}}\right). \end{array}$$

Hence, any entangled state detected by $W_{A_{1}B_{1}}⊗V_{A_{2}B_{2}}$ can also be detected by $M_{A_{1}B_{1}}⊗V_{A_{2}B_{2}}$. Therefore, $M_{A_{1}B_{1}}⊗V_{A_{2}B_{2}}$ is finer than $W_{A_{1}B_{1}}⊗V_{A_{2}B_{2}}$. □

**Theorem 1.** 
*Among all nonlinear EWs in the form of*

(2) $$W_{A_{1}B_{1}}^{\alpha }⊗V_{A_{2}B_{2}}^{\beta }=\left(\alpha I-L_{A_{1}B_{1}}\right)⊗\left(\beta I-T_{A_{2}B_{2}}\right),$$

*where $\alpha \geq \lambda_{\max}\left(L_{A_{1}B_{1}}\right)$ and $\beta \geq \lambda_{\max}\left(T_{A_{2}B_{2}}\right)$, the choice of $\alpha =\lambda_{\max}\left(L_{A_{1}B_{1}}\right)$ and $\beta =\lambda_{\max}\left(T_{A_{2}B_{2}}\right)$ yields the optimal nonlinear EW with this family.*

**Proof.**  According to Lemma 1, $W_{A_{1}B_{1}}^{\lambda_{\max}\left(L_{A_{1}B_{1}}\right)}⊗V_{A_{2}B_{2}}^{\beta }$ is finer than $W_{A_{1}B_{1}}^{\alpha }⊗V_{A_{2}B_{2}}^{\beta }$. Similarly, $W_{A_{1}B_{1}}^{\lambda_{\max}\left(L_{A_{1}B_{1}}\right)}⊗V_{A_{2}B_{2}}^{\lambda_{\max}\left(T_{A_{2}B_{2}}\right)}$ is finer than $W_{A_{1}B_{1}}^{\lambda_{\max}\left(L_{A_{1}B_{1}}\right)}⊗V_{A_{2}B_{2}}^{\beta }$. Therefore, the nonlinear EW expressed as $W_{A_{1}B_{1}}^{\lambda_{\max}\left(L_{A_{1}B_{1}}\right)}⊗V_{A_{2}B_{2}}^{\lambda_{\max}\left(T_{A_{2}B_{2}}\right)}$ is optimal among the family of EWs (2). □

From a geometric perspective, the set of separable states S forms a convex set in the Hilbert–Schmidt space of quantum states [8]. A linear EW corresponds to a supporting hyperplane of S, separating some entangled states from S. In contrast, the nonlinear EW expressed as W⊗V, constructed via tensor products, defines a quadratic hypersurface in the composite state space that can bend around the linear hyperplanes and detect entangled states inaccessible to linear EWs.

Our main optimization result in Theorem 1 admits a clear geometric meaning: the optimal nonlinear witness is achieved under weakly optimal parameters of $\alpha =\lambda_{\max}\left(L\right)$ and $\beta =\lambda_{\max}\left(T\right)$. Geometrically, this choice makes the nonlinear hypersurface tightest to the boundary of *S*, touching the separable set while maximally expanding into the entangled region. Any deviation from these parameters either shrinks the detectable entangled region or breaks the supporting property, leading to a less efficient witness. When constructing a nonlinear EW, one should first optimize each linear EW to be weakly optimal before taking the tensor product. Thus, Theorem 1 provides a clear criterion for parameter selection in experimental implementations.

## 4. Nonlinear EWs from Identical Linear EWs

Besides dealing with the optimization problem presented in Section 3, another open question is whether one can construct nonlinear EWs using identical EWs. For certain special EWs, we can rigorously prove that identical EWs do not yield nonlinear EWs. Let

$$F=\sum_{i,j=0}^{d-1}|ij\rangle \langle ji|$$

be the flip operator acting on $C^{d}⊗C^{d}$. Then, *F* is an EW from [8]. The following theorem shows that $F_{A_{1}B_{1}}⊗F_{A_{2}B_{2}}$ is not a nonlinear EW.

**Theorem 2.** 
*If $F_{A_{1}B_{1}}=F_{A_{2}B_{2}}$, then the operator expressed as $F_{A_{1}B_{1}}⊗F_{A_{2}B_{2}}$ cannot detect any entangled state.*

**Proof.**  According to [25], any state ρ on $C^{d}⊗C^{d}$ admits the decomposition of

(3) $$\rho =\sum_{i=1}^{n}p_{i}|\xi_{i}\rangle \langle \xi_{i}|⊗|\eta_{i}\rangle \langle \eta_{i}|,$$

where $p_{i},i=1,2,\ldots ,n,$ are real coefficients. By direct calculation, we obtain

(4) $$\begin{array}{cc} \; & \mathrm{tr}\left(F_{A_{1}B_{1}}⊗F_{A_{2}B_{2}}\rho_{A_{1}A_{2}}⊗\rho_{B_{1}B_{2}}\right)\\ \; & =\sum_{i,j}p_{i}p_{j}\mathrm{tr}\left(F_{A_{1}B_{1}}|\xi_{i}\rangle {\langle \xi_{i}|}_{A_{1}}⊗|\xi_{j}\rangle {\langle \xi_{j}|}_{B_{1}}\right)\mathrm{tr}\left(F_{A_{2}B_{2}}|\eta_{i}\rangle {\langle \eta_{i}|}_{A_{2}}⊗|\eta_{j}\rangle {\langle \eta_{j}|}_{B_{2}}\right)\\ \; & =\sum_{i,j}p_{i}p_{j}\langle \xi_{i}\xi_{j}|F_{A_{1}B_{1}}|\xi_{i}\xi_{j}\rangle \langle \eta_{i}\eta_{j}|F_{A_{2}B_{2}}|\eta_{i}\eta_{j}\rangle \\ \; & =\sum_{i,j}p_{i}p_{j}{\left|\langle \xi_{i}|\xi_{j}\rangle \right|}^{2}{\left|\langle \eta_{i}|\eta_{j}\rangle \right|}^{2}=p^{†}\left(\left(M\circ \bar{M}\right)\circ \left(N\circ \bar{N}\right)\right)p, \end{array}$$

where $p={\left(p_{1},p_{2},\ldots ,p_{n}\right)}^{†}$ is a real vector and *M* and *N* are Hermitian matrices defined by

$$M={\left(\langle \xi_{i}|\xi_{j}\rangle \right)}_{n\times n},\;N={\left(\langle \eta_{i}|\eta_{j}\rangle \right)}_{n\times n}.$$

Here, the ∘ symbol denotes the Hadamard (entry-wise) product of matrices [26], and $\bar{•}$ denotes the complex conjugate of a matrix.

It is straightforward to verify that $M=X^{†}X$ is positive semi-definite with $X=\left(|\xi_{1}\rangle \;|\xi_{2}\rangle \;\ldots \;|\xi_{n}\rangle \right)$. Furthermore, the complex conjugate $\bar{M}$ is also positive semi-definite. According to [26], the Hadamard product $M\circ \bar{M}$ remains positive semi-definite. Similarly, $N\circ \bar{N}$ is positive semi-definite. Finally, $\left(M\circ \bar{M}\right)\circ \left(N\circ \bar{N}\right)$ is positive semi-definite as well. From (4), we get

$$\mathrm{tr}\left(F_{A_{1}B_{1}}⊗F_{A_{2}B_{2}}\rho_{A_{1}A_{2}}⊗\rho_{B_{1}B_{2}}\right)\geq 0$$

for any quantum state ρ. Therefore, $F_{A_{1}B_{1}}⊗F_{A_{2}B_{2}}$ cannot detect any entangled state. □

With respect to the Hilbert–Schmidt inner product, let ${\left\{G_{k}\right\}}_{k=1}^{d^{2}}$ denote an orthonormal basis for $C^{d\times d}$, where each basis matrix is Hermitian. According to [5], the following operator is an EW acting on $C^{d}⊗C^{d}$:

$$R=I-\sum_{k=1}^{d^{2}}G_{k}⊗G_{k}.$$

The following theorem shows that $R_{A_{1}B_{1}}⊗R_{A_{2}B_{2}}$ is not a nonlinear EW.

**Theorem 3.** 
*If $R_{A_{1}B_{1}}=R_{A_{2}B_{2}}$, then the operator expressed as $R_{A_{1}B_{1}}⊗R_{A_{2}B_{2}}$ cannot detect any entangled state.*

**Proof.**  Any state ρ on $C^{d}⊗C^{d}$ can be decomposed as in (3). Let

$$\rho_{i}=|\xi_{i}\rangle \langle \xi_{i}|,\;\sigma_{i}=|\eta_{i}\rangle \langle \eta_{i}|.$$

Then, we obtain

$$\begin{array}{cc} \; & \mathrm{tr}\left(R_{A_{1}B_{1}}⊗R_{A_{2}B_{2}}\rho_{A_{1}A_{2}}⊗\rho_{B_{1}B_{2}}\right)\\ \; & =\sum_{i,j}p_{i}p_{j}\mathrm{tr}\left(\left(I_{A_{1}B_{1}}-\sum_{k}G_{k}^{A_{1}}⊗G_{k}^{B_{1}}\right)\rho_{i}^{A_{1}}⊗\rho_{j}^{B_{1}}⊗\left(I_{A_{2}B_{2}}-\sum_{k}G_{k}^{A_{2}}⊗G_{k}^{B_{2}}\right)\sigma_{i}^{A_{2}}⊗\sigma_{j}^{B_{2}}\right)\\ \; & =\sum_{i,j}p_{i}p_{j}(1-\mathrm{tr}\left(\sum_{k}G_{k}^{A_{1}}⊗G_{k}^{B_{1}}\rho_{i}^{A_{1}}⊗\rho_{j}^{B_{1}}\right)-\mathrm{tr}\left(\sum_{k}G_{k}^{A_{2}}⊗G_{k}^{B_{2}}\sigma_{i}^{A_{2}}⊗\sigma_{j}^{B_{2}}\right)\\ \; & \;+\mathrm{tr}\left(\left(\sum_{k}G_{k}^{A_{1}}⊗G_{k}^{B_{1}}\rho_{i}^{A_{1}}⊗\rho_{j}^{B_{1}}\right)⊗\left(\sum_{k}G_{k}^{A_{2}}⊗G_{k}^{B_{2}}\sigma_{i}^{A_{2}}⊗\sigma_{j}^{B_{2}}\right)\right))\\ \; & =1-\mathrm{tr}\left(\sum_{k}G_{k}^{A_{1}}⊗G_{k}^{B_{1}}\rho_{A_{1}}⊗\rho_{B_{1}}\right)-\mathrm{tr}\left(\sum_{k}G_{k}^{A_{2}}⊗G_{k}^{B_{2}}\rho_{A_{2}}⊗\rho_{B_{2}}\right)\\ \; & \;+\mathrm{tr}\left(\sum_{k,l}\left(G_{k}^{A_{1}}⊗G_{l}^{A_{2}}\right)\rho_{A_{1}A_{2}}⊗\left(G_{k}^{B_{1}}⊗G_{l}^{B_{2}}\right)\rho_{B_{1}B_{2}}\right)\\ \; & =1-\sum_{k}\left(\mathrm{tr}\left(G_{k}^{A_{1}}\rho_{A_{1}}\right)\mathrm{tr}(G_{k}^{B_{1}}\rho_{B_{1}}\right)-\sum_{k}\left(\mathrm{tr}\left(G_{k}^{A_{2}}\rho_{A_{2}}\right)\mathrm{tr}\left(G_{k}^{B_{2}}\rho_{B_{2}}\right)\right)\\ \; & \;+\sum_{k,l}\left(\mathrm{tr}\left(\left(G_{k}^{A_{1}}⊗G_{l}^{A_{2}}\right)\rho_{A_{1}A_{2}}\right)\mathrm{tr}\left(\left(G_{k}^{B_{1}}⊗G_{l}^{B_{2}}\right)\rho_{B_{1}B_{2}}\right)\right)\\ \; & =1-\mathrm{tr}\left(\rho_{A_{1}}^{2}\right)-\mathrm{tr}\left(\rho_{A_{2}}^{2}\right)+\mathrm{tr}\left(\rho_{A_{1}A_{2}}^{2}\right)\geq 0, \end{array}$$

where $\rho_{A_{i}}$ (resp. $\rho_{B_{j}}$) denotes the reduced density matrix of $\rho_{A_{1}A_{2}}$ (resp. $\rho_{B_{1}B_{2}}$), and the last inequality is taken from [27]. Therefore, the operator expressed as $R_{A_{1}B_{1}}⊗R_{A_{2}B_{2}}$ cannot detect any entangled state. □

Theorems 2 and 3 demonstrate that the self-tensor products of two representative linear witnesses—the flip operator *F* and the basis-derived witness *R*—fail to detect any entangled state. As we are unable to provide a rigorous theoretical proof for this general phenomenon, we state it below as an open problem.

**Problem 2.** 
*Does there exist a linear EW W acting on $C^{d}⊗C^{d}$ such that its tensor product with itself, i.e., $W_{A_{1}B_{1}}⊗W_{A_{2}B_{2}}$, can detect some entangled state?*

## 5. Conclusions

In this work, we investigated the optimization and self-tensoring construction of nonlinear entanglement witnesses. We first showed that the fineness of linear EWs does not transfer to their nonlinear tensor-product counterparts via an explicit counterexample. For the canonical family of nonlinear witnesses expressed as (αI−L)⊗(βI−T), we rigorously proved that the optimal nonlinear witness is uniquely given by weakly optimal parameters of $\alpha =\lambda_{\max}\left(L\right)\;\mathrm{and}\;\beta =\lambda_{\max}\left(T\right).$ Second, we analytically demonstrated that self-tensoring of two representative linear EWs (the flip operator *F* and the basis-derived witness *R*) yields no valid nonlinear witness.

It will be interesting to solve Problem 2 for arbitrary linear EWs in future work. Furthermore, a deeper theoretical understanding of nonlinear EWs is essential to provide a more robust foundation for their practical applications.

## Data Availability

Data is contained within the article.
